# Effectiveness of Servo-Assistive Robotic Rollator (RT.2) Among Older Adults Living in the Community

**DOI:** 10.1093/geroni/igaa057.625

**Published:** 2020-12-16

**Authors:** Takashi Yamanaka, Kiwami Kidana, Maiko Mizuki, Toshihumi Matsui

**Affiliations:** 1 The University of Tokyo, Tokyo, Japan; 2 Oouchi Hospital, Tokyo, Japan

## Abstract

Older adults tend to need assistance for ambulation with the progression of aging or when suffering from diseases. With technological advances, servo-assistive robotic rollators are available besides canes and walkers to assist disabled older adults. This study aimed to investigate the appropriate person and conditions for using a servo-assistive robotic rollator and its effects. Participants were 10 older adults living in the community (80.5±9.7 years; 4 males and 6 females) who used a servo-assistive robotic rollator (RT.2). After evaluating their physical (body composition, diseases, care need level, and SF-36), mental (MMSE, GDS-15, and WHO-5), and living conditions, they began using the device in daily life. We evaluated their ways of using it and the effects of its use through our observation and their self-report. Participants suffered from a stroke, spinal bone fracture, Parkinson’s disease, osteoarthritis of the knees, or optic neuromyelitis. At the study’s onset, cognitive impairment (MMSE<23/30), depressive states (GDS-15≧5), decreased grip strength, and decreased muscle mass (InBody S10) was found in one, three, six, and one participant, respectively. Most participants had a clear purpose for using it, such as going outside by foot or maintaining muscle strength. During the three-month observation period, no participant fell while using it. Some participants used it in a rehabilitation program at home, while others used it in daily life and went to several places with its assistance. Servo-assistive Robotic Rollators enabled older adults with difficulty in ambulation to walk outside safely and provided a greater opportunity to participate in society.

